# Differentiation of Pseudoprogression from True Progressionin Glioblastoma Patients after Standard Treatment: A Machine Learning Strategy Combinedwith Radiomics Features from T_1_-weighted Contrast-enhanced Imaging

**DOI:** 10.1186/s12880-020-00545-5

**Published:** 2021-02-03

**Authors:** Ying-Zhi Sun, Lin-Feng Yan, Yu Han, Hai-Yan Nan, Gang Xiao, Qiang Tian, Wen-Hui Pu, Ze-Yang Li, Xiao-Cheng Wei, Wen Wang, Guang-Bin Cui

**Affiliations:** 1 Department of Radiology and Functional and Molecular Imaging Key Lab of Shaanxi Province, Tangdu Hospital, Air Force Medical University, 569 Xinsi Road, Xi’an, 710038 Shaanxi China; 2 Student Brigade, Air Force Medical University, Xi’an, 710032 Shaanxi China; 3 GE Healthcare, Shanghai, 210000 China

**Keywords:** Glioblastoma, MRI, Pseudoprogression, Radiomics, Texture feature, Machine learning

## Abstract

**Background:**

Based on conventional MRI images, it is difficult to differentiatepseudoprogression from true progressionin GBM patients after standard treatment, which isa critical issue associated with survival. The aim of this study was to evaluate the diagnostic performance of machine learning using radiomics modelfrom T_1_-weighted contrast enhanced imaging(T_1_CE) in differentiating pseudoprogression from true progression after standard treatment for GBM.

**Methods:**

Seventy-sevenGBM patients, including 51 with true progression and 26 with pseudoprogression,who underwent standard treatment and T_1_CE, were retrospectively enrolled.Clinical information, including sex, age, KPS score, resection extent, neurological deficit and mean radiation dose, were also recorded collected for each patient. The whole tumor enhancementwas manually drawn on the T_1_CE image, and a total of texture 9675 features were extracted and fed to a two-step feature selection scheme. A random forest (RF) classifier was trained to separate the patients by their outcomes.The diagnostic efficacies of the radiomics modeland radiologist assessment were further compared by using theaccuracy (ACC), sensitivity and specificity.

**Results:**

No clinical features showed statistically significant differences between true progression and pseudoprogression.The radiomic classifier demonstrated ACC, sensitivity, and specificity of 72.78%(95% confidence interval [CI]: 0.45,0.91), 78.36%(95%CI: 0.56,1.00) and 61.33%(95%CI: 0.20,0.82).The accuracy, sensitivity and specificity of three radiologists’ assessment were66.23%(95% CI: 0.55,0.76), 61.50%(95% CI: 0.43,0.78) and 68.62%(95% CI: 0.55,0.80); 55.84%(95% CI: 0.45,0.66),69.25%(95% CI: 0.50,0.84) and 49.13%(95% CI: 0.36,0.62); 55.84%(95% CI: 0.45,0.66), 69.23%(95% CI: 0.50,0.84) and 47.06%(95% CI: 0.34,0.61), respectively.

**Conclusion:**

T_1_CE–based radiomics showed better classification performance compared with radiologists’ assessment.The radiomics modelwas promising in differentiating pseudoprogression from true progression.

## Background

Glioblastoma multiforme (GBM) is the most common primary malignant brain tumor in adults. Although maximal safe surgical resection followed by concurrent chemoradiotherapy (CCRT) with temozolomide (TMZ) and adjuvant TMZ has been a standard treatment, the prognosis of GBM patients is still very poor. Specially, the median overall survival ranges from 14 to 16 months, and the 2-year survival rate is only 26–33% [1, 2]. To improve this situation, the early and accurate diagnosis of postoperative progression has become very critical because it can directly influence the optimal therapy schemeselection associated with patient survival.

However, the pseudoprogression is a treatment-related change within 12 weeks after the completion of CCRT, including inflammation, radiation effects, ischemia and increased vascular permeabilityand contrast enhancement on MR imaging [3]. Both the true progression and pseudoprogression exhibit progressive enlargement and new enhancement within the radiation field. It is also difficult to differentiate them with conventional MRI sequences because pseudoprogression can mimic true progression in terms of tumor location, morphology, and enhancement patterns [4]. However, their treatments and prognosis are completely different [5]. Generally, pseudoprogressionshows better outcomes and overall survival without invasive treatment [2]. According to the Response Assessment in Neuro-Oncology (RANO) criteria [3], the current strategy to distinguish pseudoprogression from true progression heavily depends on continuous follow-up MRI examinations. Where, it may take several months to obtain a reliable diagnosis, resulting in the delay or inappropriate management of progressed GBM patients [6]. Moreover, studies by Ellington et al. [7] have shown that once tumor recurrence occurs, there is no consensus on its treatment standard. Then, even if the most aggressive treatment is adopted, it is expected that there will be no significant survival benefit. Therefore, it is crucial to develop an effective method to differentiate pseudoprogression from true progression as early as possible.

Although advanced MR imaging techniques, including diffusion-weighted imaging (DWI), perfusion-weighted imaging e.g. arterial spin labeling (ASL), dynamic contrast-enhanced MRI(DCE) anddynamic susceptibility contrast perfusion MRI (DSC) andmagnetic resonance spectroscopy (MRS), have been demonstrated to be promising in differentiating pseudoprogression from true progression, there are still limitations for them.First, the lesions were measured on the basis of a single slice region of interest(ROI)or the hot-spot method, leading to theincompleteassessment of tumors [8, 9]. Second, the limited image information applied in these studies cannot fully address tumor heterogeneity. Third, excessive parameters and time-consuming post-processing limit their clinical applications [10, 11]. Besides, advanced sequences highly depend on the performance of the scanner and are not available in all hospitals.Thus, it is urgent to develop a user-friendly protocol for the early and comprehensive differentiation ofpseudoprogression from true progression.

Recently, the term radiomics,byextracting a large number of quantitative image features combined with machine learning algorithms, radiomics can provide information that is difficult to perceive by visual inspection to guide clinical decision-making, has attracted increased attention in the medical field, especially in tumor research for diagnosis, staging and prognosis [12–15]. Theradiomics strategy hasalso been used to identify pseudoprogression and true progression [16–18]. However, most of them were largely focused on advanced MR techniques, andthe varied post-processing models, varied interpretation and uniform standards for evaluation restricted their clinical applications. In contrast, T_1_CE is widelyused in almost all hospitalsfor thediagnosis and follow-upof GBM patients. Thus, developing an effective T_1_CE based radiomics model to differentiate pseudoprogression and true progressionwill have great potential in clinic.

In this study, we aimed to evaluate the diagnostic power of T_1_CE imaging radiomics-based machinelearning in differentiating pseudoprogression from true progression inGBM patients after standard treatment.The diagnostic power of radiomics model was further compared with that of radiologists’ assessment.

## Methods

### Patient population

This study was approved by our institutional review board, and the requirement for informed consent was waived based on its retrospective nature. One hundred thirty-one pathologically confirmed primary GBM patients were retrospectively enrolled from May 2014 to February 2017 in Tangdu hospital.

The inclusioncriteria were as follows: (1) GBM patients underwent gross total resection or subtotal resection of the lesion; (2) routine MRI was performed within 48 h after surgery, including T_1_-weighted imaging (T_1_WI) and contrast-enhanced T_1_WI; (3) the patients underwent standard treatment (CCRT with TMZ and six cycles of adjuvant TMZ after surgery); (4) the patients underwent a second round of MR imaging within 2 months after CCRT with TMZ, and the third follow-up MRI examination was obtained at 6 months after CCRT [19]; (5) the patients did notreceivecorticosteroidtreatment3 days before each MRI examination; (6) the patients had new or enlarged enhancement within the radiation field on the second follow-up MR images; and (7) thepatientswere confirmed to havetrue progression or pseudoprogression through pathology after the second surgery or clinical radiologic follow-up.

Fifty-four patients were excluded for the following reasons: (1) absence of new or enlarged enhancement at the end of radiation therapy with concurrent TMZ (*n* = 15); (2) lack ofstandardized treatment schedules after surgery (*n* = 10); (3)poor image quality or motion artifacts (*n* = 11); and (4) lack ofcomplete clinical radiological follow-up or pathological evidence (*n* = 18).

**Fig. 1:**
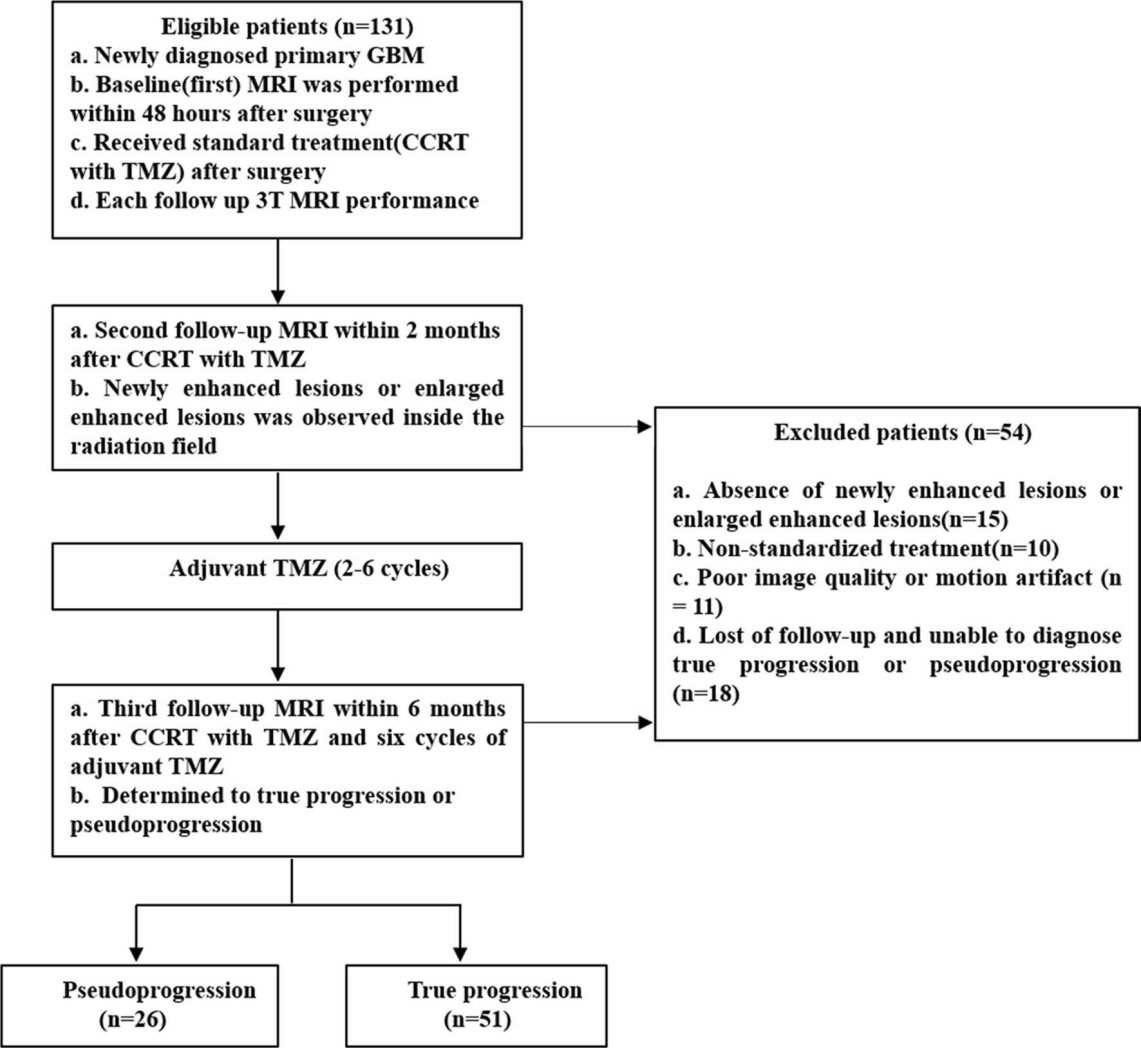
Flow chart of patient enrollment

Finally, 77 patients were included and confirmed to have true progression (*n* = 51) or pseudoprogression (*n* = 26). Thirteen patients with true progression and 2 patients with pseudoprogression were confirmed by pathology of the reoperation samples. The other 2 patients died of GBM-related complications within 9 months and were also classified intothe true progression group. The other patients with true progression (*n* = 36) or pseudoprogression (*n* = 24) according to the RANO criteria [3]. The details of the patient enrollment are shown in Fig. 1.

### Image Acquisition

The MRI protocol was performed on a 3.0 T MRI scanner (MR750, GE Healthcare, and Milwaukee, Wisconsin, USA) with an 8-channel head coil (General Electric Medical System). Preoperative and the follow-up MR images were collected including axial T_1_-weighted imaging (T_1_WI), T_2_-weighted imaging (T_2_WI), fluid-attenuated inversion recovery (FLAIR) and T_1_-weighted contrast-enhanced imaging (T_1_CE).

The scanning parameters were as follows: axial T_1_WI(TR/TE, 1750 ms/24 ms; matrix size, 256 × 256; FOV,24 × 24 cm; number of excitations (NEX), 1; slice thickness, 5 mm; and gap, 1.5 mm),axial T_2_WI(TR/TE, 4247 ms/93 ms; matrix size, 512 × 512; FOV, 24 × 24 cm; NEX, 1; slice thickness, 5 mm; and gap, 1.5 mm), sagittal T_2_WI (TR/TE, 4338 ms/96 ms; matrix size,384 × 384; FOV, 24 × 24 cm; NEX, 2; slice thickness, 5 mm; and gap, 1.0 mm), and axial FLAIR (TR/TE, 8000 ms/165 ms; matrix size, 256 × 256; FOV, 24 × 24 cm; NEX, 1; slice thickness, 5 mm; and gap, 1.5 mm). Finally, a contrast-enhanced T_1_-weighted spin-echo sequence was acquired in the transverse, sagittal, and coronal planes after intravenous administration of 0.1 mmol/kg gadodiamide (Omniscan; GE Healthcare, Co., Cork, Ireland).

### Segmentation of the volume of interest(VOI)

**Fig. 2:**
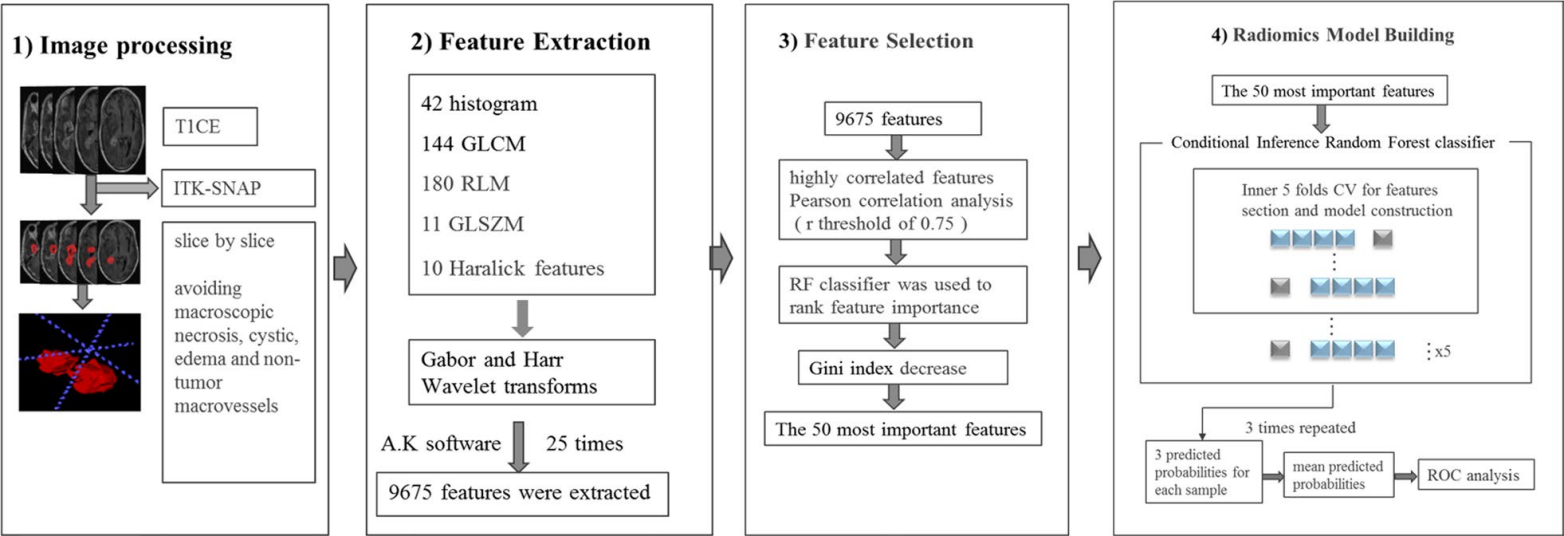
Workflow of image processing and machine learning

The research pipeline, including image preprocessing, feature extraction, feature selection and radiomics model building is depicted in Fig. 2.Two neuroradiologists (L.F.Y., with 12 years of experience and Y.Z.S., with 10 years of experiencein neuro-oncology imaging) independently reviewed all images. A third senior neuroradiologist (G.B.C., with 25 years of experience in brain tumor imaging) re-examined the images and determined the finalclassificationwhen inconsistencies existed between the two neuroradiologists. In assessing whether the lesion progressed after complete resection, the preoperative image features of the tumor would affect the results. Thus, the preoperative image features of the tumor were also observed and characterized based on the criteria outlined in Additional file 1: Table S1.

**Table 1:** Clinical characteristics of patients

Variable	Total	Pseudoprogression	True progression	P value
No. of patients	77	n = 26	n = 51	NA
Gender				
Male	40	12 (46.2%)	28 (54.9%)	0.482*
Female	37	14 (53.8%)	23 (45.1%)	
Age				
Mean	49.1 ± 10.5	47.1 ± 10.2	50.1 ± 10.4	0.230**
Karnofsky Performance Scale Score				
≤ 80	36	11 (89.3%)	25 (98.9%)	0.635*
> 80	41	15 (10.7%)	26 (1.1%)	
Surgery				
Subtotal resection	17	5 (29.4%)	12 (70.6%)	0.776*
Gross total resection	60	21 (35%)	39 (65%)	
Neurological Deficit				
No	44	16 (36.4%)	28 (63.6%)	0.633*
Yes	33	10 (30.3%)	23 (69.7%)	
Mean Radiation Dose(Gy)	59.1	59.5	58.6	0.365*

Except where indicated, data are numbers of patients

^a^Data are mean ± standard deviation

*Calculated by using the Fisher’s exact test. **Calculated by using unpaired Student t test

The difference between the groups was significant (*P* < 0 .05)

The VOIs were semi-automatically segmented by the two neuroradiologists(L.F.Y. and Y.Z.S.)using ITK-SNAP (version 3.6, http://www.itk-snap.org). The VOIs covering the enhanced lesion were drawn slice by slice on T_1_CE, avoiding the regions of macroscopicnecrosis, cystic, edema and non-tumor macrovessels, at the second follow-up MR imaging within 2 months after standard treatment [20].

## Radiomics Strategy

### Feature Extraction

A series of texture featureswere involved in this study, including 42 histogram features, 11 Gy level size zone matrix (GLSZM) texture features, 10 Haralick features, 144 Gy level co-occurrence matrix (GLCM) texture features and 180 run-length matrix (RLM) texture features of the original images. The after 25 times Gabor and Haarwavelettransforming. Then, a total of 9675 features were extracted from the T_1_CE images using Analysis-Kinetics (A.K., GE Healthcare) software.The aforementioned features were used here because they were found to be relevant for distinguishing glioma grades in our previous study[14].

### Feature Selection

After normalization, the highly redundant and correlated features were subjected to a two-step feature selection procedure. First, highly correlated features were eliminated using Pearson correlation analysis, with an*r* threshold of 0.75. Then, a random forest (RF) classifier consisting of a number of decision trees was used to rankthe feature importance. Specially, each node in the decision trees is a condition on a single feature, designed to split the dataset into two and similar response values will end up in the same set. The measurement based on which the (locally) optimal condition is determinedis called impurity. When training a tree, how much each feature decreases this weighted impurity in the tree can be computed. Furthermore, for a forest, the impurity decrease ofeach feature can be averaged across the trees, and then used to rank the features, i.e. features importance. In our study, the Gini impurity decrease was used as the criterion to evaluate the feature importance for feature selection.

### Radiomics Model Building

After feature ranking, the 50 most important features were fed into a conditional inference RF classifier for model fitting [21]. The synthetic minority oversampling technique (SMOTE) strategy was used to address the data imbalance issue [22].Five-fold cross validation method was employed for tuning the hyperparameter and performed 3 times to avoid bias and overfitting as much as possible. Then these results were averaged to get the final performance.

The accuracy, sensitivity and specificityof the receiver operating characteristic (ROC)were computed to evaluate the constructed radiomics model.

### Radiologists’assessment

To compare the efficacies of radiologists’ assessment and radiomics modelin differentiating pseudoprogression from true progression, the images were also evaluated by three junior neuroradiologists (Q.T., G.X. and Y.H., with 8, 7 and 7 years of experience in neuroradiology, respectively) using the second follow-up MR images when new or enlarged enhanced lesions were observed within the radiation field.The neuroradiologists were blinded to the clinical information but were aware that the tumors showedeither pseudoprogression or true progression, without knowing the exact category each patient fell in. Each readers independently assessed only the T_1_CE images and recorded a final diagnosis using a 4-point scale (1 = definite pseudoprogression; 2 = likely pseudoprogression; 3 = likely true progression; and 4 = definite true progression) [23].

### Statistics

For comparisons of the differences in clinical characteristics between thepseudoprogression and true progression groups, Fisher’s exact test or thechi-square test wasused for the categorical variables, and unpaired Student’s t test was used for continuous variables. These were performed by using SPSS 20.0 software (SPSS Inc., Chicago, IL, USA). *P* value < 0.05 was considered to indicate statistical significance.

Radiomics model construction was performed using R version 3. 4. 2 (R Foundation for Statistical Computing). The ‘RF’,‘caret’ and ‘unbalanced’R packages were used for feature selection and SMOTE, respectively. Thediagnostic performance of the radiomics model was assessedby using the accuracy, sensitivity, specificity.The samevalues of the three readers for differentiating pseudoprogression from true progression were also calculated and compared with the radiomicsmodel.

## Results

### Patient Characteristics and Qualitative MR Assessment

The patient characteristics are summarized in Table 1. The study group consisted of 40 men and 37 women with a mean age of 49.1 ± 10.5 years (range 17–76 years).The symptoms of these patients included headache and vomiting (61.0%; 47 of 77 patients), epilepsy (18.2%; 14 of 77), physical dysfunction (20.8%; 16 of 77) and others (31.1%; 24 of 77). None of the pretreatment clinical characteristics, including sex, age, Karnofsky Performance Status (KPS) score, resection extent, neurological deficit and mean radiation dose, showed significant differencein differentiating pseudoprogression from true progression.

In addition, the diagnostic powers of preoperative image features in differentiatingpseudoprogression from true progression were summarized in Additional file 1: Table S2. The side of the tumor exhibited statistically significant (*P* = 0.023), and the location of the tumor had a tendency towards statistical significancebetween-group difference (*P* = 0.053).

**Table 2:** Statistical differences of radiomic features determined by using RF classifier between pseudoprogression and true progression

Feature	Gini Importance	True progression		Pseudoprogression		*p* value
		Median	Interquartile range	Median	Interquartile Range	
Feature1	3.73	0.998	0.995–0.999	0.996	0.993–0.999	< .001
Feature2	2.91	1.30 × 10^–5^	2.0 × 10^–6^–6.8 × 10^–5^	3.39 × 10^–5^	7.34 × 10^–6^–1.19 × 10^–4^	< .001
Feature3	2.08	3.0 × 10^–13^	1.04 × 10^–14^–4.2 × 10^–12^	5.59 × 10^–13^	1.26 × 10^–13^–7.91 × 10^–12^	.079
Feature4	2.08	− 0.20	− 1.21–0.83	− 0.58	− 1.79–1.09	< .001
Feature5	1.98	1.14 × 10^4^	1725.0–72,802.4	2.03 × 10^4^	9098.51–56,899.20	.015
Feature6	1.53	3.32 × 10^–4^	1.44 × 10^–4^–7.51 × 10^–4^	4.65 × 10^–4^	1.71 × 10^–4^–7.57 × 10^–4^	< .001
Feature7	1.45	16.22	1.20–241.05	37.06	11.14–254.88	.137
Feature8	1.42	221.32	14.89–5051.62	349.15	89.95–5227.03	.765
Feature9	1.39	5.25 × 10^–6^	2.45 × 10^–7^–2.04 × 10^–5^	6.44 × 10^–7^	2.43 × 10^–7^–8.2 × 10^–6^	.828
Feature10	1.32	5.35 × 10^8^	2.20 × 10^7^–1.64 × 10^11^	2.34 × 10^9^	1.85 × 10^8^–9.06 × 10^10^	.374
Feature11	1.25	4.84 × 10^–5^	1.3 × 10^–5^–1.96 × 10^–4^	7.3 × 10^–5^	5.74 × 10^–6^–1.75 × 10^–4^	.008
Feature12	1.25	14.49	1.08–342.81	35.44	2.47–189.35	.244
Feature13	1.24	− 2393.65	− 61,416.60–36,264.10	− 1.26 × 10^4^	− 152 × 10^5^–5.76 × 10^4^	.015
Feature14	1.09	1.5 × 10^–13^	5.05 × 10^–15^–8.51 × 10^–9^	2.72 × 10^–13^	8.2 × 10^–14^–1.83 × 10^–11^	.445
Feature15	1.07	1.8 × 10^–5^	1.22 × 10^–6^–7.55 × 10^–5^	2.8 × 10^–5^	5.75 × 10^–6^–1.08 × 10^–4^	.005
Feature16	1.01	0.998	0.994–0.999	0.996	0.993–0.999	< .001
Feature17	0.93	3.27 × 10^–5^	− 3.14 × 10^–4^–7.18 × 10^–4^	1.47 × 10^–4^	− 5.47 × 10^–4^–4.69 × 10^–4^	.050
Feature18	0.93	− 744.67	− 1.68 × 10^4^–1.03 × 10^4^	748.24	− 11,634.40–18,560.10	.138
Feature19	0.89	0.12	1.91 × 10^–4^–8.93	0.14	5.95 × 10^–4^–5.43	.197
Feature20	0.82	0.55	0.35–0.66	0.56	0.50–0.73	.028
Feature21	0.81	1.3 × 10^–13^	1.20 × 10^–14^–2.97 × 10^–12^	2.52 × 10^–13^	5.05 × 10^–14^–2.07 × 10^–9^	.161
Feature22	0.76	1.83 × 10^9^	6.18 × 10^7^–7.80 × 10^10^	6.48 × 10^9^	7.02 × 10^8^–9.28 × 10^10^	.048
Feature23	0.75	0.998	0.994–0.999	0.997	0.994–0.998	.256
Feature24	0.74	0.998	0.994–1.000	0.998	0.997–0.999	.347
Feature25	0.73	5.1 × 10^–12^	1.53 × 10^13^–4.33 × 10^10^	1.44 × 10^–11^	1.17 × 10^–12^–3.16 × 10–8	.141
Feature26	0.72	1.73 × 10^–4^	− 2.99 × 10^–4^–7.68 × 10^–4^	1.69 × 10^–4^	3.13 × 10^–6^–7.69 × 10^–4^	.060
Feature27	0.71	3.72 × 10^–4^	2.19 × 10^–4^–1.06 × 10^–3^	4.43 × 10^–4^	3.16 × 10^–4^–1.04 × 10^–3^	.006
Feature28	0.70	7.47 × 10^3^	999.18–41,102.90	1.16 × 10^4^	1287.42–25,000.80	.111
Feature29	0.69	− 342.35	− 4559.64–8392.05	672.42	− 8078.63–28,881.70	.208
Feature30	0.68	− 1.02 × 10^3^	− 5065.29–1823.32	− 600.96	− 2031.27–3107.47	.125
Feature31	0.66	6.80 × 10^8^	2.59 × 10^7^–1.87 × 10^10^	2.12 × 10^9^	3.88 × 10^7^–3.44 × 10^11^	.103
Feature32	0.65	843.33	160.59–1046.56	753.13	258.74–1333.93	.147
Feature33	0.62	− 9.8 × 10^–5^	− 5.9 × 10^–4^–3.22 × 10^–4^	− 8.60 × 10^–5^	− 3.36 × 10^–4^–1.4 × 10^–4^	.799
Feature34	0.62	967.43	69.37–6660.59	2441.03	149.65–10,040.5	.002
Feature35	0.60	5.83 × 10^–6^	1.51 × 10^–6^–1.89 × 10^–5^	8.91 × 10^–6^	2.43 × 10^–6^–2.95 × 10^–5^	.015
Feature36	0.58	1.69 × 10^4^	9790.15–26,645.1	18,893.30	13,895.80–32,379.50	.008
Feature37	0.58	1.99 × 10^–4^	− 3.4 × 10^–4^–8.7 × 10^–4^	2.87 × 10^–4^	− 7.65 × 10^–5^–1.44 × 10^–3^	.060
Feature38	0.53	− 467.89	− 3.00 × 10^4^–1.79 × 10^4^	799.64	− 35,322.10–20,325.90	.575
Feature39	0.53	4.95 × 10^–9^	1.34 × 10^–9^–1.60 × 10^–8^	5.83 × 10^–9^	5.38 × 10^–10^–3.45 × 10^–8^	.037
Feature40	0.53	14.17	− 5.15 × 10^3^–1.25 × 10^4^	− 939.69	− 27,364.50–5113.09	.026
Feature41	0.52	− 259.96	− 16,902.50–9521.71	1264.01	− 10,087.90–6781.62	.121
Feature42	0.52	− 1.20	− 2.52–0.10	− 0.81	− 2.07–0.20	.043
Feature43	0.52	2.22	0.30–12.95	2.76	1.54–5.43	.536
Feature44	0.52	9.53 × 10^10^	1.10 × 10^10^–4.10 × 10^12^	2.30 × 10^11^	4.82 × 10^9^–7.26 × 10^12^	.023
Feature45	0.52	1.04 × 10^5^	2.21 × 10^4^–5.96 × 10^5^	1.40 × 10^5^	37,793.10–516,907.00	.025
Feature46	0.52	3.30 × 10^5^	1.23 × 10^5^–2.56 × 10^6^	4.29 × 10^5^	1.27 × 10^5^–1.39 × 10^6^	.505
Feature47	0.51	9.1 × 10^–14^	9.09 × 10^–14^–9.42 × 10^–15^	1.96 × 10^13^	4.65 × 10^–14^–8.41 × 10^–13^	.110
Feature48	0.50	0.51	0.39–0.64	0.48	0.38–0.62	.074
Feature49	0.49	6.27 × 10^–7^	5.39 × 10^–8^–4.22 × 10^–6^	1.37 × 10^–6^	1.71 × 10^–7^–4.76 × 10^–6^	.005
Feature50	0.49	− 4.9 × 10^–3^	− 0.55–0.50	0.06	− 0.68–0.63	.414

Feature relevance was assessed by using mean decrease in Gini index–based feature importance

P values are adjusted for false-discovery rate by using Benjamini–Hochberg method. 1–50 features are the same as in Fig. 4

**Fig. 3:**
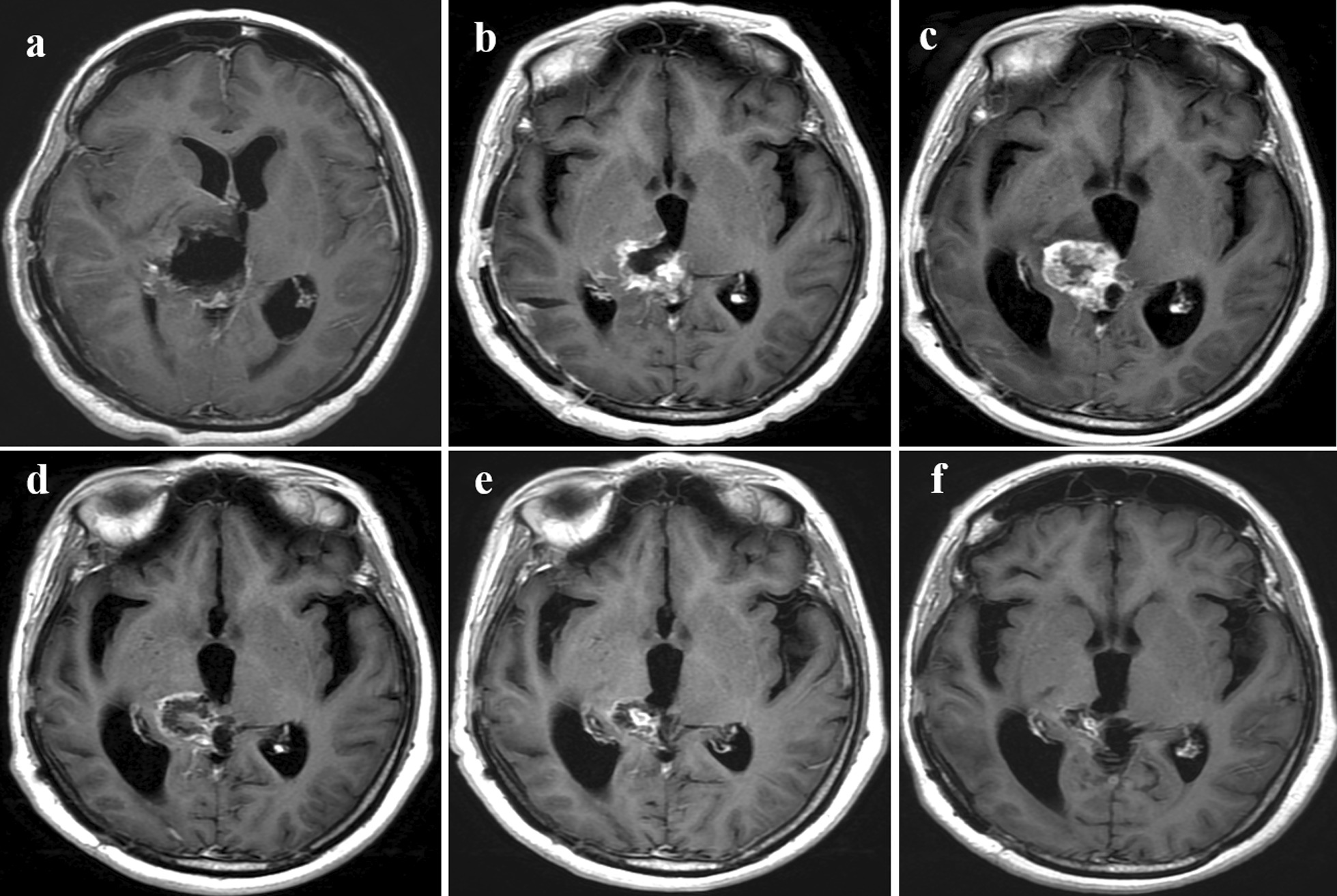
T_1_CE images showing pseudoprogression in a 45-year-old female patient with GBM. (a) Postoperative MRI (within 48 h after surgery) showing complete tumor resection. (b) Three days before CCRT, MRI showed mild enhancement of the cavity walls denoting surgical trauma-related changes. (c) Two months after CCRT, enhancement markedly increased. After CCRT and at the (d) 6-, (e) 8- and (f) 11-month follow-ups, the follow-up MR images demonstrated that the degree of lesion enhancement was reduced and the extent of enhancement was reduced. (CCRT: concurrent chemoradiotherapy)

**Fig. 4:**
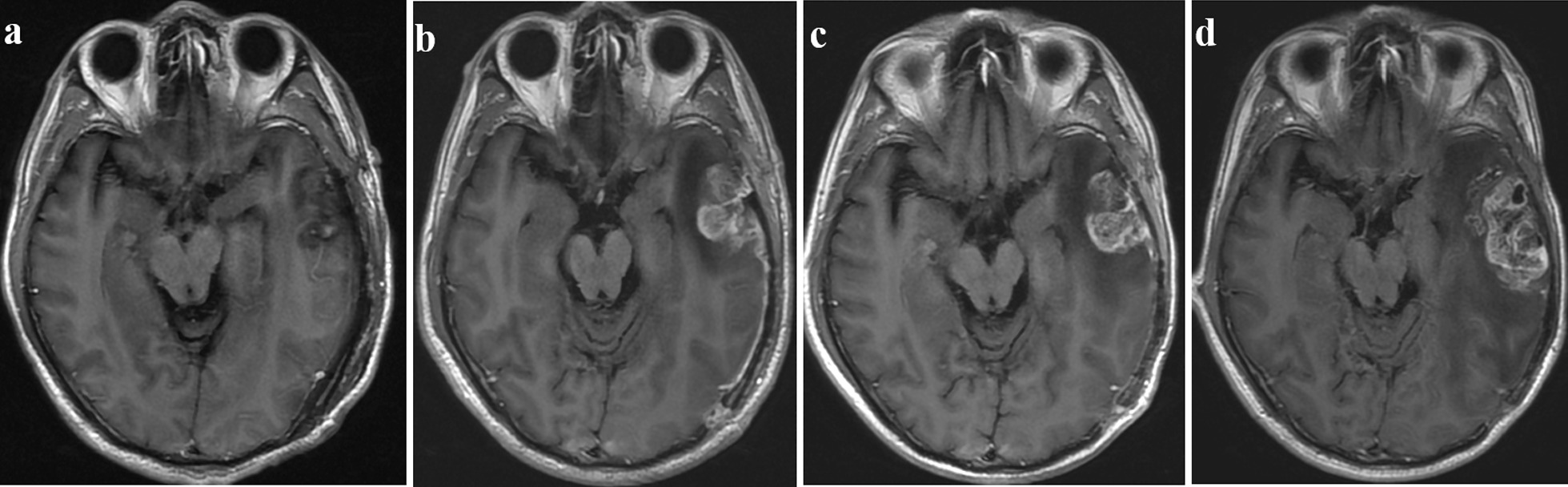
T_1_CE images showing true progression in a 48-year-old male patient with GBM. (a) Postoperative MRI (within 24 h after surgery) showed that the tumor was completely resected. (b) Two months after CCRT, the new enhancement disappeared. After CCRT and at the (c) 6-and (d) 9-month follow-ups, the follow-up MR images demonstrated that the extension of the enhanced lesion increased. Recurrence was confirmed by second surgical pathology. (GBM: glioblastoma multiforme)

Figures 3 and 4 demonstrate representative patients withpseudoprogression and true progression onT1CE imaging, respectively. The pseudoprogression case (Fig. 3), in the absence of more interventions, showed a strengthened extent of the lesion and a reduced degree of enhancement. The case of true progression (Fig. 4) showed a marked increase in the extent of the enhanced lesions, which was confirmed by secondary surgical pathology as tumor recurrence.

### Quantitative MR Texture Analysis

**Fig. 5:**
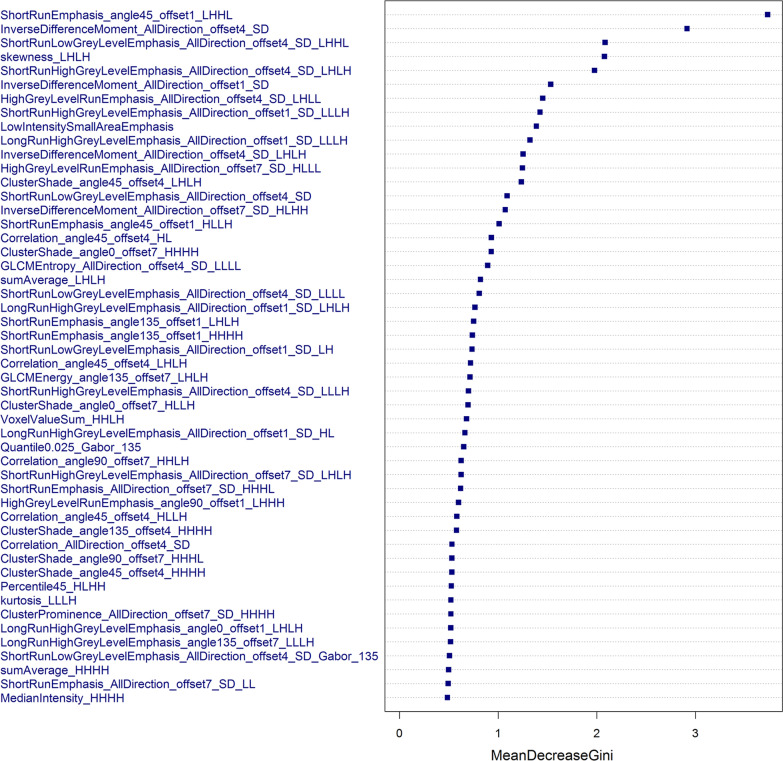
Feature importance plot showing the mean decrease in Gini impurity. Features that most reduced the Gini impurity were those that resulted in the least misclassifications

Figure 5 depicts the relative importance of the top 50 featuresbased on the Gini index. In the present study, 92% (*n* = 46) of the key features in the radiomics model were wavelet features. Twenty-two of the top 50 texture features had significant differences between the true progression group and the pseudoprogression group (Table 2).

These optimal features included1 GLSZM texture feature, 6 histogram texture features, 19 GLCM texture featuresand 24 RLM texture features. The details of the optimal feature subsets are provided in Additional file 1: Table S3.The RLM texture features accounted forthe highest proportion of the top 50 features, among which Short Run Emphasis_angle45_offset1_LHHL was the most relevant feature and was significantly lower in patients with true progression than in patients with pseudoprogression (Table 2). The GLCM texture feature was the second most dominant featurecomputed from T_1_CE (Fig. 5)and was significantly higher in patients with pseudoprogression than in patients with true progression (Table 2). The histogram feature and GLSZM texture feature were the least relevantof the top 50 features. Skewness_LHLH and low intensity small area emphasis were the fourth and ninth most relevant features (Fig. 5) and were significantly lower in patients with true progression than in patients with pseudoprogression (Table 2). Low intensity small area emphasisindicated that hypointense zones were more likely to be present inpseudoprogression patients. The above results indicated that lesions with a relatively homogenous appearance were associated with pseudoprogression.

The optimal performance was obtained by using an RF classifier trained with 50 trees. The RF classifier achieved an ACC of 72.78% (95% confidence interval [CI]: 0.45, 0.91) for differentiating pseudoprogression from true progression,with a sensitivity of 78.36% (95% CI: 0.56,1.00), and a specificity of 61.33% (95% CI: 0.20,0.82)(Table 3).

**Table 3:** Diagnostic performances of the radiomics model for differentiating pseudoprogression from true progression versus the radiologists’ assessment

	**ACC**	**Sensitivity**	**Specificity**
Radiomics	72.78%(95% CI: 0.45,0.91)	78.36%(95% CI: 0.56,1.00)	61.33%(95% CI: 0.20,0.82)
Radiologist 1	66.23%(95% CI: 0.55,0.76)	61.50%(95% CI: 0.43,0.78)	68.62%(95% CI: 0.55,0.80)
Radiologist 2	55.84%(95% CI: 0.45,0.66)	69.25%(95% CI: 0.50,0.84)	49.13%(95% CI: 0.36,0.62)
Radiologist 3	55.84%(95% CI: 0.45,0.66)	69.23%(95% CI: 0.50,0.84)	47.06%(95% CI: 0.34,0.61)

### Comparison of the diagnostic performance between theradiomicsmodeland the radiologists’ assessment

Table 3 showed the comparison of the diagnostic performance of the radiomicsmodel and the radiologists’ assessment using the sameT_1_CE image data.The accuracy, sensitivity and specificity of three radiologists’ assessment were 66.23% (95% CI: 0.55, 0.76), 61.50% (95% CI: 0.43, 0.78) and 68.62% (95% CI: 0.55, 0.80); 55.84%(95% CI: 0.45, 0.66), 69.25% (95% CI: 0.50, 0.84) and 49.13% (95% CI: 0.36, 0.62); 55.84% (95% CI: 0.45, 0.66), 69.23% (95% CI: 0.50, 0.84) and 47.06% (95% CI: 0.34, 0.61), respectively.In comparing the diagnostic performance, theACC,sensitivityand specificityof the radiomics model were significantly higher than those of the three radiologists’ assessment.

**Fig. 6:**
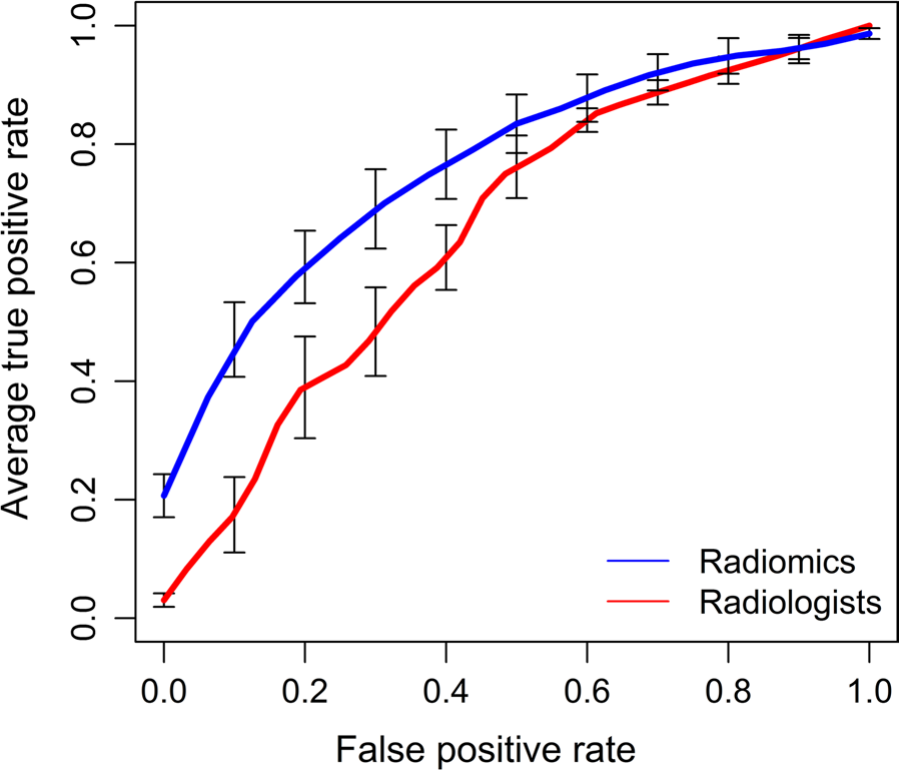
Graph shows receiver operating characteristic curve for radiomics model in differentiating pseudoprogression from true progression versus the radiologists’ assessment in GBM patients after CCRT

The ROC curve in Fig. 6 indicated that the radiomics model hasbetter diagnostic performance than the radiologists’ assessment.

## Discussion

In this study, none of the pretreatment clinical characteristics showed significant difference between the two groups.In addition, according to the results of preoperative imaging characteristics analysis,only the side of the tumor was statistically significantand the location of the tumor had a tendency towards statistical significance between two groups (Additional file 1: Table S2). The results may be related to the small sample size and data imbalance, we will observe the results in future research.

The ability of quantitative radiomics features based on T_1_CE imagesto differentiate pseudoprogression from true progressionin patients with GBM after CCRTwas investigated in the current study.When combined with RF classifier, the radiomics model achieved relatively gooddiagnosis performance with higher ACC (72.78%) and sensitivity (78.36%) than radiologists’ assessment.

Regarding the top 50 most important features selectedby using the Gini index as a metric,most of them were RLM (*n* = 24) and GLCM (*n* = 19) features. The RLM mainly reflects the roughness and directionality of the texture.The GLCM reflects the intensity of the spatial distribution[24].The histogram features (*n* = 6) and GLSZM texture feature (*n* = 1) were also played an important role in identifying pseudoprogression and true progression.The ninth important feature of low intensity small area emphasis indicated that hypointense zones were more likely to be present in pseudoprogression patients. Previous literature reports have shown that low intensity small area emphasis may reflect fibrinoid necrosis, oligodendroglial injury and glial cell hyperplasia [11, 25]. The higher the valuewas the greater the probability of pseudoprogression, which appears as a low-signal region. On the contrast,recurrent GBM was characterized by vascular proliferation and a disrupted blood–brain barrier, leading to the high signal intensity in the T_1_CE image caused by contrast agent leakage [11, 26]. Above texture features mainly reflect the tumor heterogeneity and complexity of components based on voxel-based changes in grayscale[27]. Specially, the Haralick features were not in the top 50 features, whichprobablysuggested that these two groups of features were not effective in distinguishing pseudoprogression from true progression and needed to be verified in future research.

Moreover, it can be observed that, in our study, 92% (*n* = 46) of the key features in the radiomics model were Gabor filtered wavelet features.The use of high-dimensional feature helps to improve the performance of the model.This finding demonstrates thatthe wavelet features can provide more information about the tumor invisible to the eye, so as to better assess treatment response [28, 29].

Previous studies have used low-dimensional features coupled witha few pieces of information from multi-parametric histograms[16]orSVM classification based on DCE MRI to differentiate pseudoprogression from true progression [30]. Although these studies achieved good results in differentiating pseudoprogression from true progression inGBM patients with standard treatment, there were still certain disadvantages. First, the samples and quantitative features in previous studies were relatively small, especially the relatively small number of pseudoprogression patientswithout proper handling, which might have overshadowed their statistical results [16]. Second, previous studies were mostly based on advanced MR sequences that were of much equipment dependent and may hamper its application in some primary hospitals.

To the best of our knowledge, there is no published study in the literature comparing the radiomics model with radiologists’ assessment for distinguishing pseudoprogression from true progression. In our study, the radiomics model demonstrated betterdiagnostic performance than the radiologists’ assessment. It suggested that our radiomics model may have the potential to help clinicians make an earlier judgment for patients in whom a “wait and see” approach may be the most appropriate.

### Study limitations

Several limitations of the current study should be addressed. First, the sample size was still small,so there may be a risk of overfitting. In order to solve the problem of small sample size and overfitting risk, we adopted the following methods: 1)25 times Gabor and wavelet transformations were performed on the features extracted from the original images. 2) five-fold cross validation was employed for tuning the hyperparameter and was performed 3 times to avoid bias and overfitting as much as possible.3) the SMOTE strategy was used to address the data imbalance issue, especially the sample size of pseudoprogression was relatively small. Moreover, Bum-Sup Jang et al. built a radiomics model by machine learning algorithm differentiating pseudoprogression from true progression with the total amount of sample they used was 78 cases [31]. In the future, a much larger dataset needs to be investigated to validate the robustness and reproducibility of the currently proposed radiomics model.Second, molecular alterations, such as isocitrate dehydrogenase (IDH) mutation and oxygen 6-methylguanine-DNA methyltransferase (MGMT) promoter methylation status, were not included in this study. The recently published 2016 WHO classification of brain tumors incorporated genetic parameters into the classical histopathological findings. These genetic alterations have prognostic implications in terms of survival and response to therapies [32, 33]. These indicators will be included in future studies.

## Conclusion

In conclusion, our study showed that theproposed radiomics model based on conventional T_1_CE had stable diagnostic efficacy and performed better than the radiologists’ assessment in the early differentiation ofpseudoprogression from true progressioninGBM patients after CCRT. The radiomics model may assist clinicians in the early, accurate judgment of recurrence and provide a novel tool to guide individual treatment strategies for GBM patients.

## Supplementary Information

**Additional file 1.** Details of the preoperative image features and top 50 importance features for differentiating pseudoprogression from true progressionthe.

## Data Availability

The datasets used and/or analyzed during the current study are available from the corresponding author upon reasonable request.

## Abbreviations

AUC: Area Under the Curve
ADC: Apparent Diffusion Coefficient
ASL: Arterial Spin Labeling
DCE: Dynamic Contrast Enhancement
DSC: Dynamic Susceptibility Contrast
DWI: Diffusion-weighted Imaging
GLCM: Gray-level Cooccurrence Matrix
GLSZM: Gray Level Size Zone Matrix
KPS: Karnofsky Performance Status
MGMT: O^6^-methylguanine-DNA Methyltransferase
ROC: ReceiverOperating Characteristic
ROI: Region of Interest
RLM: Run-length Matrix
RF: Random Forest
SVM: Support Vector Machine
SMOTE: Synthetic Minority Oversampling Technique
T1CE: T_1_-weighted Contrast-enhanced Imaging
VOI: Volume of Interest
