# Supplementary material for: Differentiation of Pseudoprogression from True Progressionin Glioblastoma Patients after Standard Treatment: A Machine Learning Strategy Combinedwith Radiomics Features from T1-weighted Contrast-enhanced Imaging
Source: BMC Med Imaging. 2021 Feb 3;21:17. doi: 10.1186/s12880-020-00545-5 (PMC7860032; doi:10.1186/s12880-020-00545-5)
Supplement: Supplementary file 1 — Additional file 1. Details of the preoperative image features and top 50 importance features for differentiating pseudoprogression from true progressionthe. [file 12880_2020_545_MOESM1_ESM.docx]

**Supplementary Table S1 Imaging features definition**

| **Imaging Features** | **Scoring value** | **Classification criteria** |
| --- | --- | --- |
| **Tumor location** | Type I  Type II  Type III  Type IV | Tumor in which the contrast-enhancing lesion contacts both the SVZ and the cortex.  Tumor contacts the SVZ but not the cortex.  Tumor contacts the cortex but not the SVZ.  Tumor contacts neither the SVZ nor the cortex. |
| **Tumor cross midline** | Yes/No | Tumor is limited to the unilateral cerebral hemisphere.  Tumor crosses the brain midline and extends into contralateral cerebral hemisphere. |
| **Necrosis or Cyst** | No  Mild  Severe | No necrosis or cyst within the tumor.  Necrosis or cyst affecting ≤ 50% of the tumor.  Necrosis or cyst affecting > 50% of the tumor. |
| **Side of tumor** | Right  Center/Bilateral  Left | Side of lesion epicenter irrespective of whether the lesion crosses into the contralateral hemisphere. |
| **Proportion enhancing** | Nodular  Patchy  Ringlike | Largest focal contrast-enhancement diameter of tumor ≤ 1.5 cm.  Maximum contrast-enhancement diameter of tumor＞1.5 cm.  Cystic necrosis with peripheral enhancement. |
| **PTE** | No  Minor  Major | No convincing edema.  PTE extending <1 cm from tumor margin.  PTE extending ≥1 cm from tumor margin. |
| **Hemorrhage** | Yes/No | Intrinsic hemorrhage anywhere in the tumor matrix.  Any intrinsic foci of low signal on T2WI or high signal on T1WI. |
| **Size,(cm)** | <5  ≥5 | The maximum diameter of the tumor is <5 cm.  The maximum diameter of the tumor is ≥5 cm. |

Note: Location classification was based on the spatial relationship of the contrast-enhancing lesion to the subventricular zone. (SVZ = subventricular zone). PTE= Peritumoral edema.

**Supplementary Table S2 Imaging features results**

| **Imaging Features** | **Pseudoprogression** | | **True progression** | **Total** | **P-value*** |
| --- | --- | --- | --- | --- | --- |
| **Tumor location, n (%)**  Type I  Type II  Type III  Type IV | 9/26(35)  2/26(8)  10/26(38)  5/26(19) | 13/51(25)  18/51(35)  12/51(24)  8/51(16) | | 22/77(29)  20/77(26)  22/77(28)  13/77(17) | 0.053 |
| **Tumor cross midline, n (%)**  Yes  No | 6/26(23)  20/26(77) | 5/51(10)  46/51(90) | | 11/77(14)  66/77(86) | 0.168 |
| **Necrosis or Cyst, n (%)**  No  Mild  Severe | 3/26(11)  14/26(54)  9/26(35) | 7/51(14)  18/51(35)  26/51(51) | | 10/77(13)  32/77(42)  35/77(45) | 0.286 |
| **Side of tumor, n (%)**  Right  Center/Bilateral  Left | 15/26(58)  7/26(27)  4/26(15) | 24/51(47)  5/51(10)  22/51(43) | | 39/77(50)  12/77(16)  26/77(34) | 0.023 |
| **Proportion enhancing, n (%)**  Nodular  Patchy  Ringlike | 3/26(12)  10/26(38)  13/26(50) | 4/51(8)  21/51(41)  26/51(51) | | 7/77(9)  31/77(40)  39/77(51) | 0.864 |
| **PTE, n (%)**  No  Minor  Major | 2/26(8)  8/26(31)  16/26(61) | 1/51(2)  15/51(29)  35/51(69) | | 3/77(4)  23/77(30)  51/77(66) | 0.451 |
| **Hemorrhage, n (%)**  Yes  No | 7/26(27)  19/26(73) | 8/51(16)  43/51(84) | | 15/77(19)  62/77(81) | 0.361 |
| **Size, (cm), n (%)**  <5  ≥5 | 17/26(65)  9/26(35) | 25/51(49)  26/51(51) | | 42/77(55)  35/77(45) | 0.228 |

Note: The P values were calculated from the Fisher’s exact test.

**Supplementary Table S3**: **Details of the top 50 importance features for differentiating pseudoprogression from true progression**

| Groups | Gray Level  Size Zone Matrix  (GLSM) | Histogram | Grey Level  Co-occurrence Matrix (GLCM) | Run-length matrix  (GLRLM) |
| --- | --- | --- | --- | --- |
| Number | 1 | 6 | 19 | 24 |
|  |  |  |  |  |
| Radiomics features  T1CE imaging | LowIntensitySmallAreaEmphasis | skewness_LHLH; LowIntensitySmallAreaEmphasis;  VoxelValueSum_HHLH;  Quantile0.025_Gabor_135; Percentile45_HLHH; kurtosis_LLLH;  MedianIntensity_HHHH | InverseDifferenceMoment_AllDirection_offset4_SD; InverseDifferenceMoment_AllDirection_offset1_SD; InverseDifferenceMoment_AllDirection_offset4_SD_LHLH; ClusterShade_angle45_offset4_LHLH; InverseDifferenceMoment_AllDirection_offset7_SD_HLHH;Correlation_angle45_offset4_HL; ClusterShade_angle0_offset7_HHHH; GLCMEntropy_AllDirection_offset4_SD_LLLL; sumAverage_LHLH; Correlation_angle45_offset4_LHLH; GLCMEnergy_angle135_offset7_LHLH; ClusterShade_angle0_offset7_HLLH; Correlation_angle90_offset7_HHLH; Correlation_angle45_offset4_HLLH; ClusterShade_angle135_offset4_HHHH; Correlation_AllDirection_offset4_SD; ClusterShade_angle90_offset7_HHHL; ClusterShade_angle45_offset4_HHHH; Cluster Prominence_All Direction_offset7_SD_HHHH; sumAverage_HHHH; | ShortRunEmphasis_angle45_offset1_LHHL; ShortRunLowGreyLevelEmphasis_AllDirection_offset4_SD_LHHL; ShortRunHighGreyLevelEmphasis_AllDirection_offset4_SD_LHLH; HighGreyLevelRunEmphasis_AllDirection_offset4_SD_LHLL; ShortRunHighGreyLevelEmphasis_AllDirection_offset1_SD_LLLH; LongRunHighGreyLevelEmphasis_AllDirection_offset1_SD_LLLH; HighGreyLevelRunEmphasis_AllDirection_offset7_SD_HLLL; ShortRunLowGreyLevelEmphasis_AllDirection_offset4_SD; ShortRunEmphasis_angle45_offset1_HLLH; ShortRunLowGreyLevelEmphasis_AllDirection_offset4_SD_LLLL; LongRunHighGreyLevelEmphasis_AllDirection_offset1_SD_LHLH; ShortRunEmphasis_angle135_offset1_LHLH; ShortRunEmphasis_angle135_offset1_HHHH; ShortRunLowGreyLevelEmphasis_AllDirection_offset1_SD_LH; ShortRunHighGreyLevelEmphasis_AllDirection_offset4_SD_LLLH; LongRunHighGreyLevelEmphasis_AllDirection_offset1_SD_HL; ShortRunHighGreyLevelEmphasis_AllDirection_offset7_SD_LHLH; ShortRunEmphasis_AllDirection_offset7_SD_HHHL; HighGreyLevelRunEmphasis_angle90_offset1_LHHH; LongRunHighGreyLevelEmphasis_angle0_offset1_LHLH; LongRunHighGreyLevelEmphasis_angle135_offset7_LLLH; ShortRunLowGreyLevelEmphasis_AllDirection_offset4_SD_Gabor_135; ShortRunEmphasis_AllDirection_offset7_SD_LL |

Note: T1CE= T1-weighted Contrast Enhanced.
